# Artificial UV Weathering of Retrieved Poly(vinyl butyral) Interlayer from Laminated Glass Waste: Effects of Residual Additives and Restabilization with Phenolic Antioxidants

**DOI:** 10.3390/ma19214751

**Published:** 2026-11-09

**Authors:** Vasilis Nikitakos, Athanasios D. Porfyris, Triantafyllia Alevizaki, Eleni Rakanta, Beatriz Yecora, Angelica Perez, Rudolf Pfaendner, Constantine D. Papaspyrides, Konstantinos G. Beltsios

**Affiliations:** 1 Laboratory of Polymer Technology, School of Chemical Engineering, National Technical University of Athens, 15780 Athens, Greece; vnikitakos@mail.ntua.gr (V.N.); ch20066@mail.ntua.gr (T.A.); kp@cs.ntua.gr (C.D.P.); 2 Research Lab of Advanced, Composites, Nanomaterials and Nanotechnology (R-NanoLab), School of Chemical Engineering, National Technical University of Athens, 15780 Athens, Greece; 3 Department of Materials Science & Engineering, School of Chemical Engineering, Zographou Campus, National Technical University of Athens, 15780 Athens, Greece; erakanta@central.ntua.gr; 4 LUREDERRA Technological Centre, Perguita Industrial Area, 31210 Los Arcos, Spain; beatriz.yecora@lurederra.es (B.Y.); angelica.perez@lurederra.es (A.P.); 5 Fraunhofer Institute for Structural Durability and System Reliability LBF, 64289 Darmstadt, Germany; rudolf.pfaendner@lbf.fraunhofer.de

**Keywords:** PVB, interlayer, recycling, stabilizers, weathering

## Abstract

**Highlights:**

- Neat unplasticized PVB is highly susceptible to photooxidative degradation.
- High-quality retrieved PVB (re-PVB) exhibited remarkable resistance to artificial UV weathering up to 1500 h.
- The superior UV durability of re-PVB is attributed to the action of residual stabilizers.
- Restabilization of re-PVB with phenolic antioxidants can reduce its UV stability.

**Abstract:**

Poly(vinyl butyral) (PVB) is widely used as an interlayer in laminated safety glass in automotive and construction applications. The increasing quantities of PVB arising from end-of-life laminated glass represent a waste stream with significant potential for recycling. This study investigates the durability of high-quality retrieved PVB (re-PVB) separated from laminated glass wastes and evaluates the effect of restabilization against UV aging. Neat unplasticized PVB (u-PVB) and re-PVB were subjected to artificial UV weathering (ISO 4892-3) for up to 1500 h and then characterized in terms of chemical structure, yellowness, melt flow behavior and molecular weight. The results showed that u-PVB exhibited pronounced photooxidative degradation, while re-PVB remained comparatively stable. UV–Vis spectroscopy revealed the presence of residual UV absorbers in re-PVB, indicating that the components of the initial stabilization system might remain functional after the end of its life. Unexpectedly, the restabilization of re-PVB with phenolic antioxidants (AOs) reduced its photooxidative stability, despite their established effectiveness against thermo-mechanical degradation in melt reprocessing. A possible antagonistic effect was suggested between the newly introduced AOs and the residual stabilization system. These findings highlight that re-PVB is a valuable waste, and restabilization should be carefully considered when designing its second service life.

## 1. Introduction

Poly(vinyl butyral) (PVB) serves as the interlayer material in laminated safety glass designed for automotive and architectural applications [1,2,3,4]. PVB is synthesized by the acid-catalyzed acetalization of poly(vinyl alcohol) reacting with butyraldehyde [5,6]. This reaction results in a random terpolymer composed of 70–89% *w*/*w* polyvinyl butyral (VB), 10–25% *w*/*w* polyvinyl alcohol (VA) and 1–4% *w*/*w* polyvinyl acetate (VAc) [7,8]. The hydroxyl groups of the vinyl alcohol part can interact with the silanol groups of polar substrates such as glass, resulting in strong adhesion [1,4,9,10]. Upon breakage of the laminated glass, glass fragments remain stuck to the PVB, thereby protecting the user. PVB is not used in its neat form as an interlayer but is formulated with various additives, particularly plasticizers such as aliphatic esters (diester, adipates or sebacates), to improve its processability, flexibility, and impact resistance [11]. Among these, triethylene glycol di(2-ethylhexanoate) (3GO) is the most commonly used plasticizer for PVB and has been frequently identified in commercial and post-consumer PVB interlayer samples [2]. In addition, stabilizers (antioxidants, UV absorbers, and hindered amine light stabilizers, HALSs) are also added in the final formulation to protect the polymer against thermo-mechanical and photooxidative degradation during processing and service life [12,13,14,15,16,17]. Consequently, the PVB interlayer represents a valuable material, owing not only to its specialty attributes but also to its complex formulation, which contains substantial amounts of plasticizers and functional additives.

The global PVB market was valued at approximately 3.6 billion USD in 2025 and is projected to reach 5.9 billion USD by 2033, corresponding to a compound annual growth rate (CAGR) of 6.7% [18]. The expansion of the PVB market is increasing primarily due to the demand for laminated safety glass, particularly in the automotive (59%) and architectural sectors (26%), which require high-performance PVB films that provide energy-efficient solutions [19]. The increasing production and widespread use of PVB in laminated glass are inevitably accompanied by growing quantities of post-consumer material reaching the end of its service life. In this context, the effective valorization of post-consumer PVB recovered from end-of-life (EoL) laminated glass is becoming increasingly important. Current laminated glass recycling practices predominantly focus on recycling the glass fraction, whereas post-consumer PVB is frequently directed primarily towards energy recovery or disposal or towards low-value secondary applications such as coatings, textiles or applied research in battery binders [20,21]. 

A major issue that can impede the closed-loop recycling of PVB arises from its strong adhesion to glass, thereby affecting the final quality of the retrieved material. Several delamination strategies have been proposed, including mechanical and chemical treatments based on acidic or alkaline media, surfactants, and dissolution processes to retrieve the polymer fraction [22,23,24,25]. The employed conditions during delamination may influence the chemical structure and properties of the retrieved PVB. For viable recycling, an advanced sorting system is needed to classify the laminated glass waste into categories (high and low quality). Following that, an innovative and efficient mechanochemical treatment for post-consumer laminated glass can be implemented in order to obtain highly pure retrieved PVB (re-PVB) with a low content of residual glass (<0.5% *w*/*w*), suitable for industrial PVB sheet reprocessing [26]. Herein, the combination of appropriate sorting and efficient delamination increases the potential of PVB recycling and provides a feasible route for its subsequent valorization again in its primary use as an interlayer. 

Nevertheless, successful valorization requires both efficient separation of PVB from laminated glass and sufficient material stability during subsequent processing and its second service life. Retrieved PVB (re-PVB) usually enters the recycling stream after a prolonged service (approximately 5–20 years) and is frequently stored outdoors before recycling. Added to that, it is exposed to additional thermal and shear stresses during melt reprocessing in mechanical recycling. These conditions can promote thermooxidative degradation, which proceeds through an autoxidation cycle via a free radical chain reaction process [27,28]. A reactive site (R*), a free radical, is formed on the polymer chain, reacting with oxygen to form a peroxy radical (ROO*), which further reacts with the polymer chain, creating hydroperoxides (ROOHs), which in turn decompose and generate new free radicals [29]. The cycle continues as these radicals propagate, leading to the further breakdown of the polymer and eventually resulting in the deterioration of the polymer’s mechanical behavior (embrittlement) and a downgrade in its macroscopical properties (color, haziness). This autoxidation cycle can occur throughout the service life of the polymer where organic compounds can interact with oxygen; therefore, stabilization before use is crucial for the polymer to inhibit this degradation. At the end of the life of the polymer, part of the stabilizers has usually been depleted; thus, appropriate restabilization with primary or secondary antioxidants may be required after the end of the life of the polymer to preserve the material’s performance during reprocessing and subsequent use [29,30,31]. In a previous study by Merinska et al., the thermooxidative degradation of re-PVB under conditions representative of melt reprocessing was shown to result in chain scission of the macromolecule [32]. Accordingly, the incorporation of specific hindered phenolic antioxidants was shown to effectively suppress this degradation, while providing sufficient stabilization for the next service life of the retrieved PVB [33].

While restabilization with antioxidants can effectively mitigate thermooxidative degradation during reprocessing, stability during melt processing represents only one aspect of the durability required for a successful second service life. As PVB interlayers are mostly used in applications involving prolonged exposure to solar radiation, photooxidative stability also represents a crucial parameter for their long-term durability. However, it is important to note that the photooxidative degradation of PVB may be limited by its integration between the glass layers in the laminated structure, which can block part of the UV radiation (mainly UV-B) and significantly restrict the oxygen availability for oxidation. The photooxidation cycle is initiated by the action of photons on the polymer and proceeds through an autocatalytic process in the presence of oxygen, analogous to thermooxidative degradation [28,29,30,31,32,33,34]. Particularly in PVB, light-absorbing carbonyl species such as macro-ketones may undergo photochemical reactions, including Norrish Type I and Type II reactions, which promote chain scission and molecular weight reduction [35,36]. Under irradiation, photolysis of hydroperoxides (ROOHs) represents an important source of radicals that can further propagate the oxidative chain reaction in PVB. V. Reinöhl et al. have pinpointed that photooxidation of PVB can cause reactions which promote the cleavage of acetal ring, the accompanied by an increase in carbonyl species [36]. Previous studies have also demonstrated that PVB undergoes photochemical degradation upon exposure to near-UV radiation, resulting in peroxide formation, chain scission, formation of carbonyl species and increased yellowness [35,36,37]. To mitigate these photooxidative processes and preserve material properties during prolonged outdoor exposure, PVB formulations may contain dedicated light stabilizers. Stabilization against photooxidation in polymers can happen mainly by UV absorbers, which absorb harmful UV radiation in the range of 300–400 nm and release it as heat [38]. The latter are usually used in combination with hindered amine light stabilizers (HALSs), which suppress photooxidative radical reactions through a nitroxyl regenerative formation that scavenges peroxy radicals, known as the Denisov cycle [34]. Although the restabilization of re-PVB with UV absorbers has previously been reported, its durability under artificial UV weathering has not been systematically investigated in the published literature [21]. To the best of our knowledge, no study has systematically evaluated the intrinsic UV durability of retrieved PVB prior to recycling. It remains unclear whether re-PVB should be considered as a depleted material waste requiring complete restabilization or whether a functional fraction of the original stabilization system remains active during its second service life. In fact, in a previous work from our group, it was clearly established that re-additivation with processing stabilizers (antioxidants, AOs) at ca. 0.3% *w*/*w* was mandatory to inhibit the suffered thermooxidative degradation upon melt reprocessing [33]. The present work further innovates by investigating the photooxidative behavior of high-quality PVB retrieved from laminated glass waste during artificial UV weathering for up to 1500 h. Particular emphasis is placed on the contribution of residual additives to the UV durability of re-PVB and their interactions with the newly added phenolic antioxidants on the photooxidative stability of re-PVB. An important knowledge gap is addressed by highlighting the need for restabilization strategies for end-of-life recovered polymers to consider both thermo-mechanical and UV durability, as well as potential synergistic, antagonistic, or neutral interactions between newly introduced stabilizers and residual additive system. Changes in chemical structure, molecular weight and processability are evaluated through ATR-FTIR spectroscopy, yellowness index, dilute solution viscometry (DSV), gel permeation chromatography (GPC) and melt flow rate (MFR) measurements. UV–Vis and ^1^H-NMR are also employed to assess the presence of residual additives in re-PVB. Overall, this paper provides new insight into the role of residual stabilizers and advances the handling of a high value waste stream such as re-PVB.

## 2. Materials and Methods

Unplasticized neat PVB (u-PVB), synthesized by American Polymer Standards Corporation (Mentor, OH, USA), and pure 3GO [triethylene glycol di(2-ethylhexanoate)] plasticizer, were both supplied by Eastman Chemical Company (Kingsport, TN, USA). Retrieved PVB flakes originating from automotive laminated glass wastes, characterized by low haze (<10%) and high transparency, were sorted within a demo multisensor sorting tool developed under SUNRISE H2020 project [39]. The flakes had a minimal residual glass content (<0.5% *w*/*w*), contained 3GO plasticizer (≈40% *w*/*w*) and were obtained by delamination of the sorted post-consumed laminated glass waste via a patented mechanochemical process [26]. Approximately 40 kg of retrieved PVB flakes were obtained and homogenized by melt processing in a co-rotating twin-screw extruder Haake Rheomex PTW16 (Thermo Fisher Scientific, Germany) with L/D = 25 at 150 °C and a screw speed of 100 rpm. The extruded material was subsequently pelletized to obtain a homogeneous feedstock and labeled as re-PVB (retrieved PVB).

**Figure 1:**
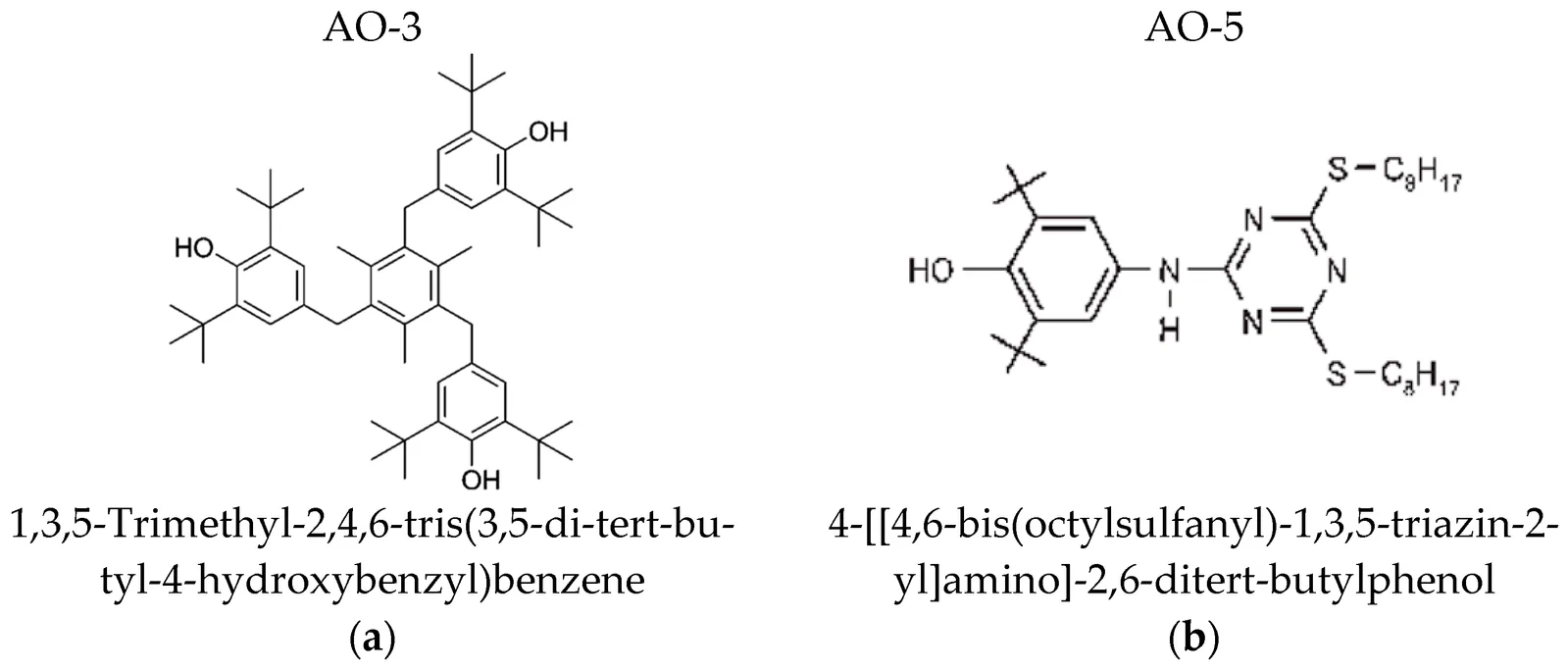
Chemical structures of the phenolic antioxidants used for the restabilization of retrieved PVB: (**a**) AO-3 and (**b**) AO-5.

A commercial plasticized (≈32% *w*/*w* 3GO) PVB grade (PVB Eastman Saflex RB41) material was included as a benchmark in selected initial characterization measurements to assess the quality of re-PVB. Selected antioxidants (AOs), AO-3 and AO-5, which are considered appropriate for the restabilization of re-PVB against thermooxidative degradation, were tested at a 0.3% *w*/*w* concentration against artificial UV weathering (Figure 1) [33]. Both antioxidants were provided by BASF Hellas S.A (Athens, Greece).

A T300B Banbury-type internal mixer (Brabender, Duisburg, Germany) was employed to homogenize the re-PVB flakes and incorporate the antioxidants. The material (50 g) was dried and then loaded in the chamber at T = 130 °C for 2 min and screw speed of 80 rpm. Upon completion of this step, the material was collected, cooled and reduced to pellets by grinding. For each formulation, four square specimens (60 × 60 × 1 mm^3^) were prepared by hot press, at 140 °C for plasticized samples and 160 °C for unplasticized samples, using a molding time of 10 min under a pressure of approximately 150 bar. One specimen per formulation was reserved for the initial characterization (t = 0 h), while the remaining specimens were subjected to artificial UV weathering for 3 sampling intervals, i.e., 500, 1000 and 1500 h.

Artificial UV weathering was conducted in a QUV Accelerated Weathering Chamber (Q-Lab) according to ISO 4892–3 standard (method A and exposure cycle 2), equipped with UVA-340 lamps (Type 1A), which closely simulate the ultraviolet part of natural solar radiation that is mainly responsible for polymer photooxidation. The cycle comprised three consecutive stages, starting with 8 h of UVA (340 nm) exposure at a spectral irradiance of 0.76 W m^−2^ nm^−1^ and a black-panel temperature of 50 ± 3 °C. The irradiation period was followed by 15 min of water spraying and 3 h 45 min of condensation, both at 50 ± 3 °C. No UV irradiation was applied during the last two stages.

Fourier transform infrared (FTIR) spectra were acquired using an Alpha II spectrometer (Bruker, Germany) coupled with an attenuated total reflectance (ATR) accessory. Spectra were collected over the wavenumber range of 400–4000 cm^−1^ at a resolution of 4 cm^−1^, with 32 stacked scans for each measurement. For aged specimens, all measurements were conducted on the surface directly exposed to UV radiation. The extent of photooxidation was also evaluated by estimating the carbonyl index (CI), defined as the ratio of the absorbance (A) at the maximum intensity of the carbonyl band found at 1709 cm^−1^ to that of the methylene C–H stretching band at 2928 cm^−1^, which was assumed to remain unaffected during weathering (Equation 1) [35].

(1) $$\mathrm{Carbonyl Index},\mathrm{CI}=\frac{{A1709}_{{\mathrm{cm}}^{-1}}}{{A2928}_{{\mathrm{cm}}^{-1}}}$$

Thermogravimetric analysis (TGA) was conducted in a Mettler Toledo TGA/DSC HT 1 (Mettler-Toledo International Inc., Greifensee, Switzerland) instrument to characterize the thermal stability and composition of the reference samples. Samples were heated from 30 to 600 °C at a constant rate of 10 °C/min under a nitrogen flow of 10 mL/min. The temperatures corresponding to plasticizer evaporation (*T*_d1_) and PVB decomposition (*T*_d2_) were identified from the respective peaks of the derivative thermogravimetric (DTG) curves, indicating the highest rates of mass loss. Additionally, plasticizer content (% *w*/*w*) and residue (% *w*/*w*) were calculated. Duplicate measurements were obtained to calculate average data values refined by the standard deviation. 

Differential scanning calorimetry (DSC) analysis was performed using a Mettler DSC 1 instrument (Mettler-Toledo International Inc., Greifensee, Switzerland). Samples underwent a heating–cooling–heating program between −20 and 210 °C, with both heating and cooling rates set to 10 °C/min. This procedure was applied to eliminate the influence of previous thermal history and impose identical conditions across the samples. A nitrogen atmosphere was maintained at a flow rate of 20 mL/min. The glass transition temperatures (*T*_g_ values) of the reference PVB samples were obtained from the 2nd heating curve.

Colorimetric analysis of the PVB specimens was conducted with a MYIRO-1 spectrophotometer (Konica Minolta, Japan), employing M2 measurement conditions and a 2° standard observer. The CIE tristimulus coordinates (X, Y, and Z) were recorded and subsequently used to determine the yellowness index (YI) in accordance with ASTM E313, as expressed in Equation (2).

(2) $$\mathrm{YI}=\frac{1.3013\times X_{\mathrm{CIE}}-1.1497\times Z_{\mathrm{CIE}}}{Y_{\mathrm{CIE}}}\times 100$$

Due to the transparency of the PVB specimens, measurements were performed on the square specimens (1 mm thickness) placed against a standard white background. Therefore, the reported YI values are influenced by the measurement configuration and are used only for comparative evaluation among the measured PVB samples. Direct comparison with YI values reported in commercial PVB datasheets is not appropriate unless equivalent measurement conditions are employed. Yellowness index (YI) was measured at three different locations on each specimen to assess the consistency of the measurements. The change in yellowness index (ΔYI) was calculated according to Equation (3) to evaluate the evolution of yellowness during artificial UV weathering.

ΔYI = |ΥΙ_0_| − |ΥΙ_1_| (3)

where *YI*_0_ and *YI*_1_ represent the average yellowness index before and after artificial UV weathering, respectively. Positive ΔYI values indicate increased yellowing, whereas negative values denote shift towards blue. 

Gel permeation chromatography (GPC) analysis was performed using an Agilent 1260 Infinity II system (Agilent Technologies, Germany), comprising a PLgel 5 μm guard column, two PLgel MIXED-D 5 μm columns (300 × 7.5 mm), and a refractive index detector (Agilent 1260 Infinity II RID, G7162A). Tetrahydrofuran (THF; ≥99.8% purity, Fisher Scientific, UK) served as both the sample dissolution solvent and the mobile phase, with elution maintained at 1 mL/min. Calibration was established using polystyrene standards (EasiVial PS-M, Great Britain) covering a molecular weight range of 162–500,000 g/mol. GPC analysis was conducted once per specimen. Accordingly, the reported molecular weight values are expressed as polystyrene-equivalent (PS-equivalent) molecular weights. 

The intrinsic viscosity (IV, [η]) of PVB samples was determined by dilute solution viscometry (DSV). For this purpose, each sample was dissolved in tetrahydrofuran (THF) solvent at a concentration of 0.5 g/dL. Flow measurements were carried out at 30 °C using an Ubbelohde-type viscometer (Cannon Instrument Company, State College, PA, USA). The efflux times of pure THF and the corresponding polymer solutions were recorded in triplicate, and the intrinsic viscosity was calculated using the single-point method described by Equation (4). Solutions that were not fully dissolved were filtered so as to isolate their insoluble fraction. Further drying in vacuo to remove solvent and weighing out, allowed the calculation of the insoluble fraction in % *w*/*w* of the initial total mass.

(4) $$\left[\eta \right]=\frac{\sqrt{1+1.5\eta_{\mathrm{sp}}}-1}{0.75C}$$

DSV measurements were performed using two independently prepared solutions obtained from the same specimen.

Melt flow rate (MFR) measurements were conducted using a Kayeness Dynisco 4004 melt indexer (Dynisco Europe GmbH, Heilbronn, Germany) in accordance with ASTM D1238-10. All tests were performed at 190 °C under loads of 2.16 kg and 10 kg for plasticized and unplasticized PVB samples, respectively. Before analysis, the specimens were dried at 60 °C for 4 h to minimize the plasticizing effect of absorbed moisture. MFR values obtained after artificial UV weathering were normalized to the initial value of each formulation (MFR_0_, at 0 h) and expressed as MFR/MFR_0_ to gauge the extent of degradation. The strands obtained from the material exiting the die of MFR were also visually examined to assess the processability and integrity of the materials after melt processing.

UV–Vis absorption spectra were recorded using a JASCO V-770 spectrophotometer (JASCO Corporation, Tokyo, Japan). The reference PVB samples and the plasticizer were separately dissolved in ethanol at 10 mg/mL, with continuous stirring until clear, uniform solutions were obtained. Following dilution, the solutions were analyzed over the wavelength interval of 300–700 nm for the polymeric materials and at 200–700 nm for the plasticizer.

Proton nuclear magnetic resonance (^1^H NMR) analysis was performed using a Bruker Avance DRX spectrometer operating at 500 MHz. Approximately 10 mg of each sample was dissolved in 0.8 mL of deuterated chloroform (CDCl_3_). Spectra were recorded at 296 K (±2 °C), and chemical shifts (δ) were expressed in parts per million (ppm). A commercial HALS, Tinuvin 770, was analyzed as reference material and compared with the spectra of u-PVB and re-PVB to investigate the possible presence of residual HALSs in the retrieved materials.

## 3. Results

### 3.1. Characterization of the Reference Materials

**Figure 2:**
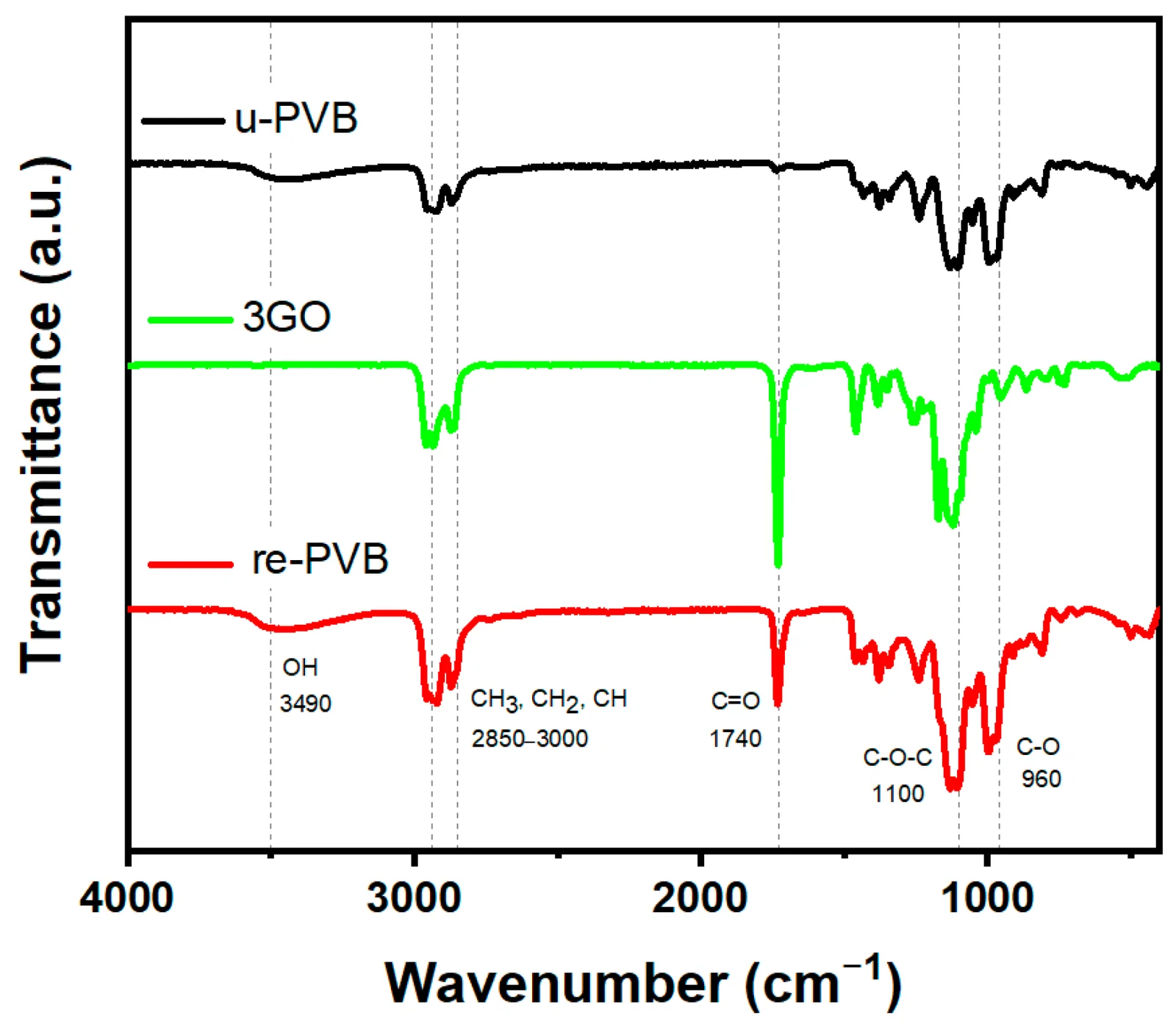
FT-IR spectra of the reference PVB materials (3GO, u-PVB, re-PVB) [33].

The initial chemical structure of the reference materials (u-PVB, 3GO and re-PVB) was evaluated by ATR-FTIR spectroscopy (Figure 2). u-PVB exhibited the characteristic peaks of an acetal ring (1–3 dioxane ring of vinyl butyral unit) at 1100 and 960 cm^−1^, corresponding to the C–O–C and C–O stretching vibrations, respectively [1,11,40]. Additionally, the broad O–H stretching band at approximately 3500 cm^−1^ was associated with residual vinyl alcohol units, resulting from incomplete acetalization of PVA. The absorption bands in the 2850–3000 cm^−1^ region are attributed to aliphatic C–H stretching vibrations of CH, CH_2_, and CH_3_ groups within the PVB structure and a relatively weak carbonyl absorption was observed around 1740 cm^−1^, which was attributed to the residual vinyl acetate units of PVB. As for the spectra of the plasticizer 3GO [triethylene glycol bis(2-ethylhexanoate)], the characteristic carbonyl peak was also found at 1740 cm^−1^, representing C=O stretching, which is indicative of an ester moiety [2]. Comparison of the u-PVB and re-PVB spectra reveals differences in intensity, mainly of the peaks in the carbonyl region (1730–1740 cm^−1^) and of ether C–O–C stretching (1100 cm^−1^). The higher intensity of these bands in re-PVB confirms the presence of the ester-based plasticizer in the polymer. Notably, re-PVB retains the characteristic absorption bands of the PVB backbone, indicating the preservation of the main polymer structure after its first service life and retrieval process.

**Figure 3:**
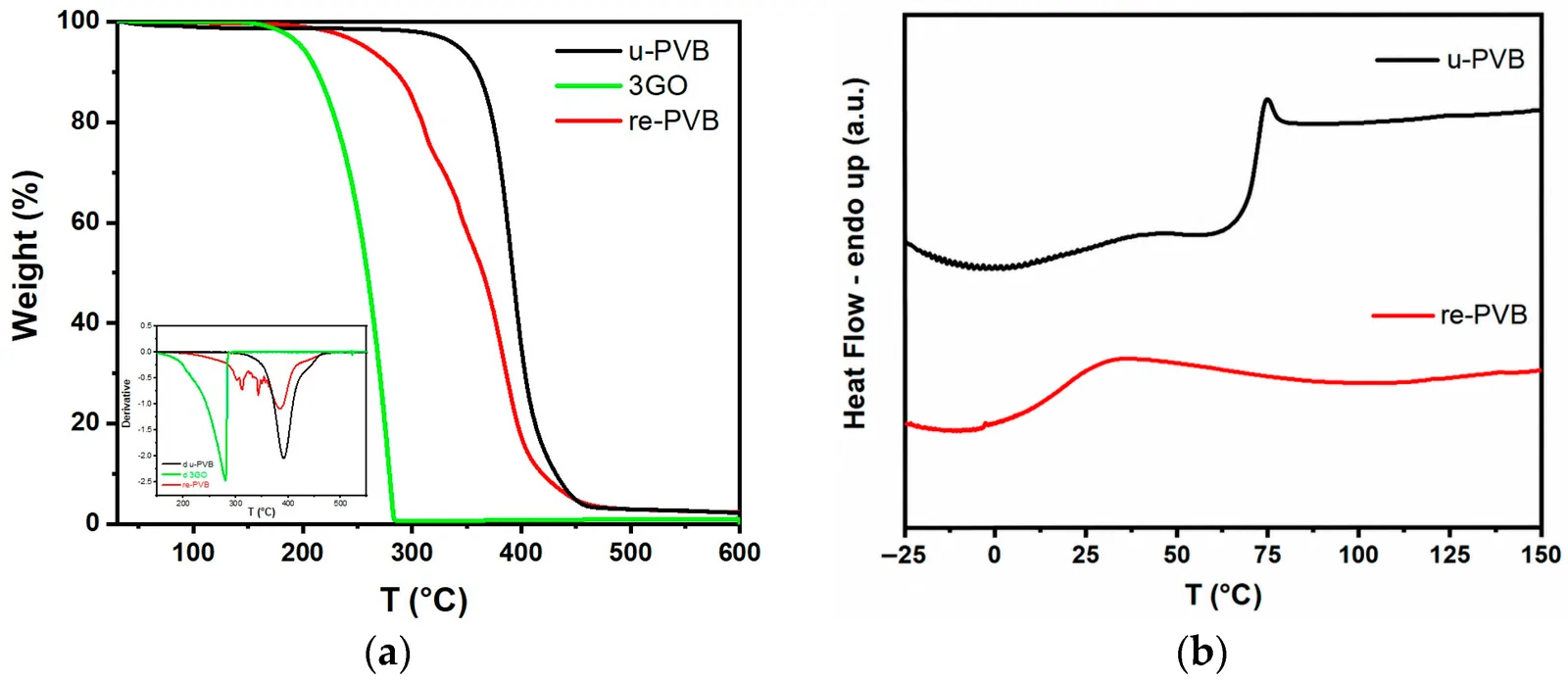
(**a**) TGA weight-loss curves of u-PVB, re-PVB, and pure 3GO plasticizer, with the corresponding derivative thermogravimetric (DTG) curves; (**b**) DSC thermograms (2nd heating) of u-PVB and re-PVB illustrating differences in their *T*_g_ values [33].

In addition, the thermal behavior of the reference materials was investigated by TGA and DSC (Figure 3, Table 1). Unplasticized PVB (u-PVB) presented a single-step mass loss curve (300–450 °C), corresponding to the decomposition of PVB, while pure 3GO exhibited substantially lower thermal stability due to its inherent volatility, with its main mass loss occurring at lower temperatures (180–290 °C). re-PVB contained both distinct decomposition regions, the first occurring at lower temperatures corresponding to the evaporation of the 3GO plasticizer from the polymeric matrix, while the second was associated with decomposition of the PVB. It is worthwhile to mention that re-PVB exhibited a slightly higher residue value than u-PVB, due to additional residues of the plasticizer and/or potential glass residues/contaminants (Table 1). The DSC analysis further demonstrated the pronounced effect of plasticization, with u-PVB having a glass-transition temperature (*T*_g_) of approximately 73 °C, whereas the plasticized re-PVB exhibited a substantially lower *T*_g_ of approximately 15–16 °C (Figure 3b), which is typical of a plasticized PVB grade material [2,41].

The initial yellowness index (YI) was also measured for the tested polymers and compared to the value of the benchmark PVB grade material (PVB Eastman Saflex RB41) (Table 2). Prior to artificial weathering, re-PVB exhibited a higher YI (40.41 ± 0.22) than both u-PVB (28.49 ± 0.86) and the benchmark PVB grade material (33.91 ± 0.04). The higher initial yellowness of re-PVB may be associated with reprocessing, the heterogeneity of different PVB grades and different plasticizer content, residual additives or slight impurities from the separation process from glass. Nevertheless, the difference between re-PVB and the commercial PVB grade material was still relatively moderate (19.2% increase), further supporting the high quality of the retrieved material and its optical characteristics being reasonably comparable to those of commercial plasticized PVB. 

**Figure 4:**
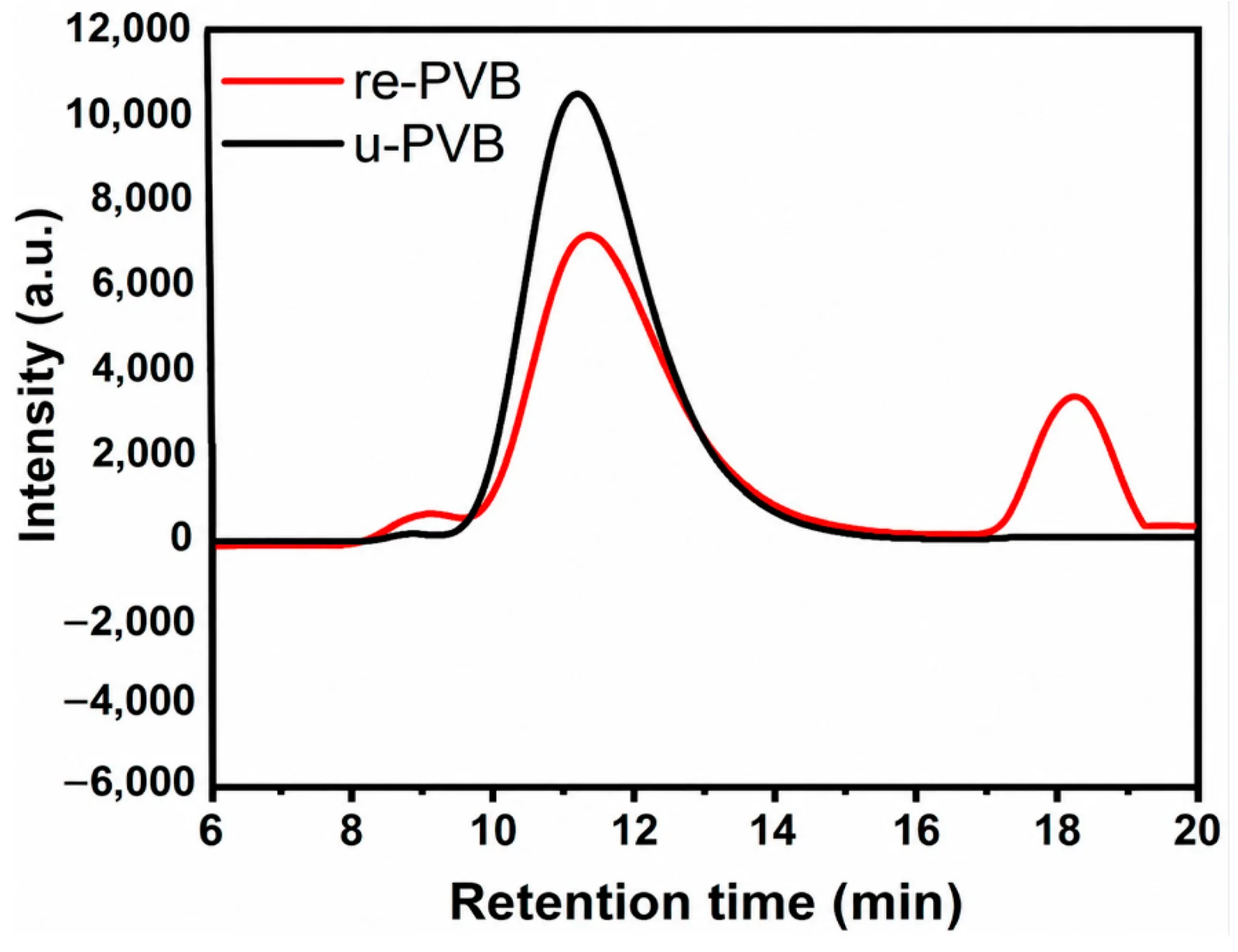
GPC chromatograms of unplasticized PVB (u-PVB) and retrieved PVB (re-PVB).

Regarding molecular weight, re-PVB exhibited a higher intrinsic viscosity (2.00 ± 0.10 dL g^−1^) than u-PVB (1.58 ± 0.01 dL g^−1^). Complementary to solution viscometry, the gel permeation chromatography (GPC) analysis revealed distinct differences between unplasticized PVB (u-PVB) and retrieved PVB (re-PVB) in terms of molecular weight and molecular weight distribution. u-PVB exhibited a relatively narrow and symmetric molecular weight distribution (Figure 4), indicative of a more uniform polymer structure. On the other hand, re-PVB displayed a broader distribution intensity (9–15 min) and the presence of an additional fraction at higher retention times (~18–19 min), which corresponds to lower molecular weight species, mainly associated with 3GO plasticizer [2]. The broader molecular weight distribution of re-PVB (PDI = 2.96) compared with u-PVB (PDI = 2.21) indicated greater molecular heterogeneity (Table 3), as expected for a post-consumer material originating from multiple sources. The GPC clarified the presence of longer polymer chains, resulting in a higher molecular weight in re-PVB (Mw = 507,000 g/mol PS-equivalent) compared to u-PVB (Mw = 463,000 g/mol PS-equivalent), in agreement with its higher intrinsic viscosity, as determined by solution viscometry. 

Lastly, the MFR of re-PVB (2.47 ± 0.15 g/10 min) was considered comparable to that of the commercial benchmark PVB grade material (2.08 ± 0.05 g/10 min), suggesting a similar melt behavior [2]. On the contrary, u-PVB, due to the absence of plasticizer, is far more viscous, thus exhibiting a much lower MFR value, even when measured with a 10 kg load.

### 3.2. Artificial UV Weathering of Reference PVB Materials

Artificial UV weathering caused markedly different responses in u-PVB and re-PVB. Under the applied weathering conditions, these changes are primarily associated with UV-A induced photooxidation. The moderate exposure temperature (50 °C) is relatively low and is mainly expected to accelerate the kinetics of photooxidative reactions rather than independently induce substantial thermal degradation. Similarly, the condensation and water-spray stages reproduce the effects of environmental moisture but are not expected to induce substantial hydrolytic degradation of the PVB backbone due to the low content of ester groups (vinyl acetate units <4% *w*/*w*) in the terpolymer [41,42]. Therefore, the changes observed for u-PVB under weathering are predominantly attributed to UV-A irradiation, with temperature and moisture acting only as complementary aging factors, simulating outdoor conditions.

**Figure 5:**
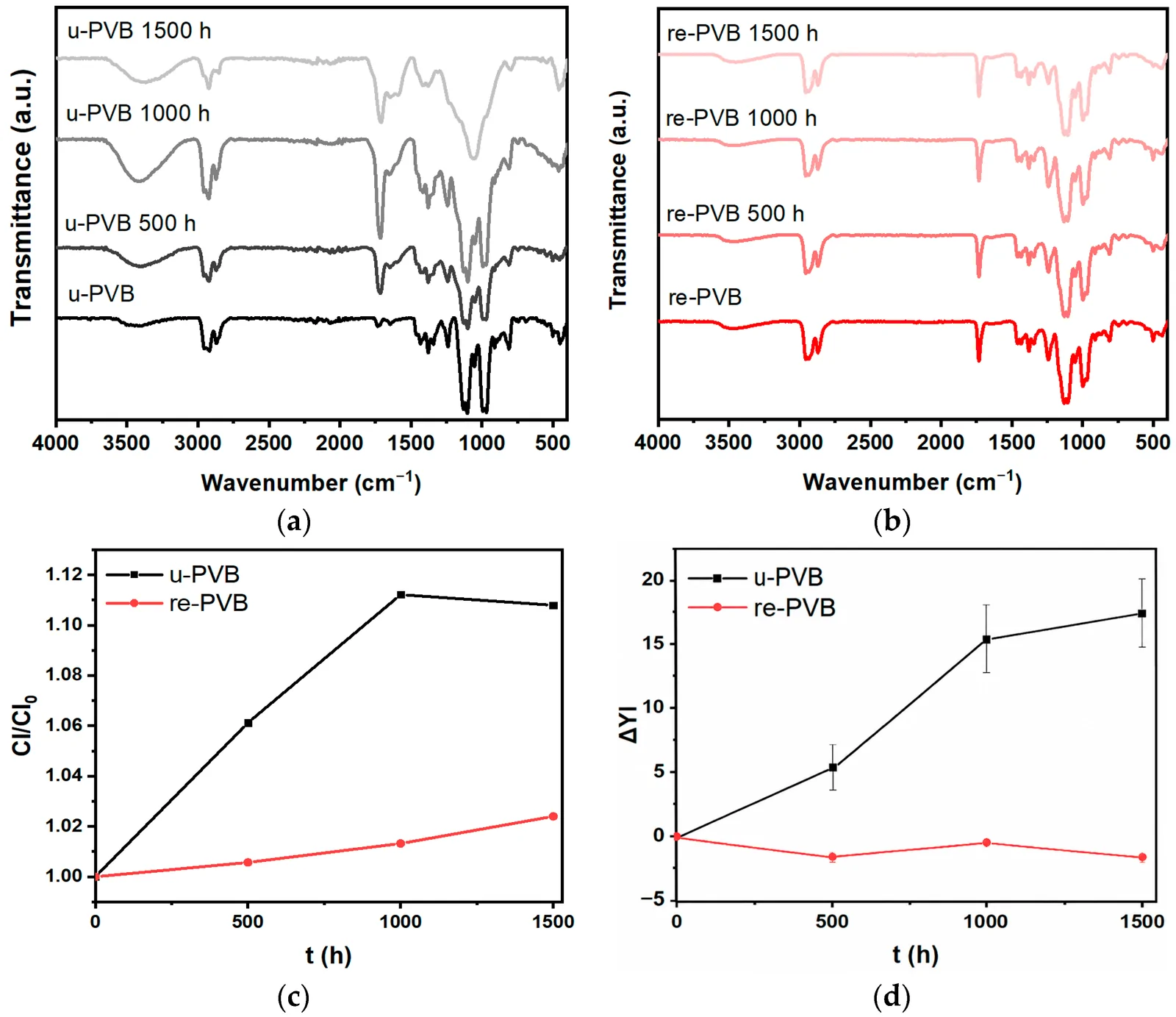
ATR-FTIR spectra of (**a**) u-PVB and (**b**) re-PVB before and after 500, 1000, and 1500 h of artificial UV weathering; (**c**) evolution of the normalized carbonyl index (CI/CI_0_) of u-PVB and re-PVB as a function of artificial UV weathering time; (**d**) changes in yellowness index (ΔYI) of u-PVB and re-PVB as a function of exposure time.

Regarding the FTIR spectra of the aged samples (Figure 5), u-PVB exhibited pronounced alterations in its chemical structure, while re-PVB remained comparatively stable throughout the whole exposure period. Progressive changes in the chemical structure of u-PVB were evidenced with increasing weathering time. After 1500 h of exposure, a pronounced increase in the carbonyl absorption band (~1700–1730 cm^−1^) was observed, indicating the formation of oxidation products such as aldehydes, ketones, and esters. The increasing absorption around 1600 cm^−1^ may also be attributed to the formation of C=C containing unsaturated structures resulting from photooxidative products from the degradation of the PVB backbone [43]. Concurrently, broadening of the O–H stretching region (~3200–3600 cm^−1^) suggests the formation of hydroxyl groups due to chain scission of the PVB backbone. Added to that, the reduction and distortion of the acetal bands (1100 and 960 cm^−1^) indicated cleavage of the acetal ring of the vinyl butyral unit (Figure 5a). These significant structural changes confirmed that prolonged UV exposure led to significant chain scission and severe photooxidative degradation of the PVB polymer matrix. UV exposure promoted the generation of a high concentration of reactive radicals and oxidative species, resulting in an intense chemical modification of the polymer structure [35,36]. During extended exposures to natural weathering or a laboratory source of ultraviolet radiation, the carbonyl content is expected to reach a plateau after a certain point [35]. Indeed, a similar behavior was observed in the present study for u-PVB, as the carbonyl index (CI) increased up to a point (1000 h), at which a peak value was reached (Figure 5c). On the contrary, re-PVB exhibited no changes in its FTIR spectra throughout the total 1500 h weathering period (Figure 5b). The characteristic absorption bands of the PVB structure remained unchanged, and there was no increase in the carbonyl index or noticeable alterations in the region of the vinyl butyral unit. This limited change in re-PVB indicated indirectly that photooxidative degradation could possibly be inhibited by residual UV stabilizers that are still active in the sample after the end of its life.

The different behaviors of u-PVB and re-PVB against artificial UV weathering was also reflected in their optical properties (Figure 5d and Figure S1a,b). u-PVB showed a gradual increase in yellowness with exposure time, reaching a high color difference (ΔYI = 17) compared to its initial color after 1500 h. In contrast, re-PVB presented a remarkable color stability throughout the weathering period, with ΔYI remaining close to zero even after 1500 h of exposure. The pronounced yellowing of u-PVB is consistent with the formation of chromophore species (ketones, esters) during photooxidative degradation, which was also noted in the FTIR spectra, whereas re-PVB demonstrated its superior color stability and resistance to artificial UV weathering, as carbonyl species were strongly limited [44]. 

**Figure 6:**
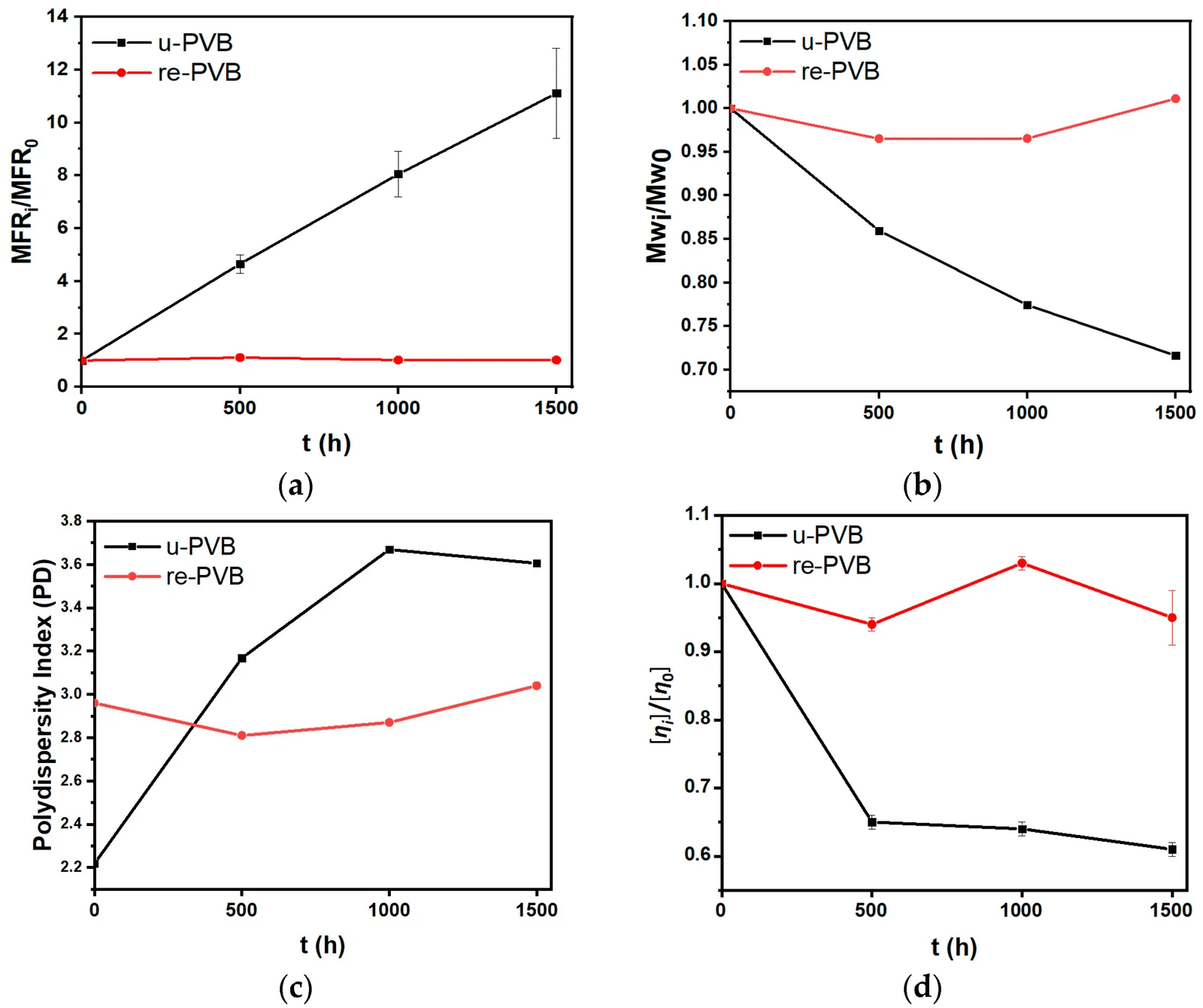
Evolution of the normalized (**a**) melt flow rate (MFR/MFR_0_) and (**b**) weight-average molecular weight (Mw/Mw_0_) from GPC for u-PVB and re-PVB during artificial UV weathering up to 1500 h. (**c**) Evolution of the polydispersity index (PDI) of u-PVB and re-PVB as a function of artificial UV weathering time for up to 1500 h. (**d**) Changes in normalized intrinsic viscosity ([η]/[η]_0_) of u-PVB and re-PVB during artificial UV weathering for up to 1500 h.

Melt flow rate provides further insight into molecular changes induced by degradation and is particularly relevant for assessing its subsequent melt reprocessability. A severe increase in the normalized MFR was observed for u-PVB (Figure 6a), reaching approximately eleven times its initial value after 1500 h of exposure. This substantial increase in the melt flow rate is indicative of a reduction in molecular weight and is consistent with chain scission during photooxidative degradation [32,33,36]. In contrast, the normalized MFR of re-PVB remained unchanged throughout the 1500 h exposure period, further confirming its high resistance to UV-induced degradation. The MFR was also used as a tool to gauge reprocessability and recyclability, judging from the macroscopic observation of the MFR strands exiting the capillary die of the melt flow indexer. With increasing UV exposure time, MFR strands of u-PVB exhibited coloring from yellow to eventually brown, whereas MFR strands of re-PVB remained clear and transparent after exiting the die at 190 °C (Figure S2a,b).

The GPC results further confirmed the different molecular response of u-PVB and re-PVB to artificial UV weathering (Figure 6b). The normalized weight-average molecular weight (Mw/Mw_0_) of u-PVB progressively decreased with exposure time, reaching approximately 0.72 after 1500 h. This molecular weight decrease, together with the pronounced increase in normalized MFR, validates that chain scission is the predominant degradation mechanism of PVB under UV weathering. In contrast, no appreciable change in the normalized Mw was observed for re-PVB at all the aging intervals. The PDI increased markedly for u-PVB from approximately 2.2 before exposure to 3.6–3.7 after prolonged weathering, showing a substantial broadening of the molecular weight distribution, whereas the PDI of re-PVB remained stable throughout the 1500 h exposure period, showcasing no alteration of its molecular weight distribution (Figure 6c). Together with GPC, dilute solution viscometry showed a consistent decrease in normalized intrinsic viscosity for u-PVB, reaching approximately 0.61 after 1500 h of UV exposure (Figure 6d). Photooxidative degradation of PVB may not be governed exclusively by chain scission but also by competing intermolecular reactions, potentially leading to branching or even crosslinked structures, consistent with previous observations from the literature [35,37]. The latter was evidenced by the solutions of degraded u-PVB samples that exhibited an insoluble fraction (gel fraction). More specifically, the gel fraction for u-PVB at 1500 h was determined at ca. 5% *w*/*w*, further supporting the previous macroscopical observation. On the contrary, no insoluble fraction was evidenced in the aged samples of re-PVB. 

**Figure 7:**
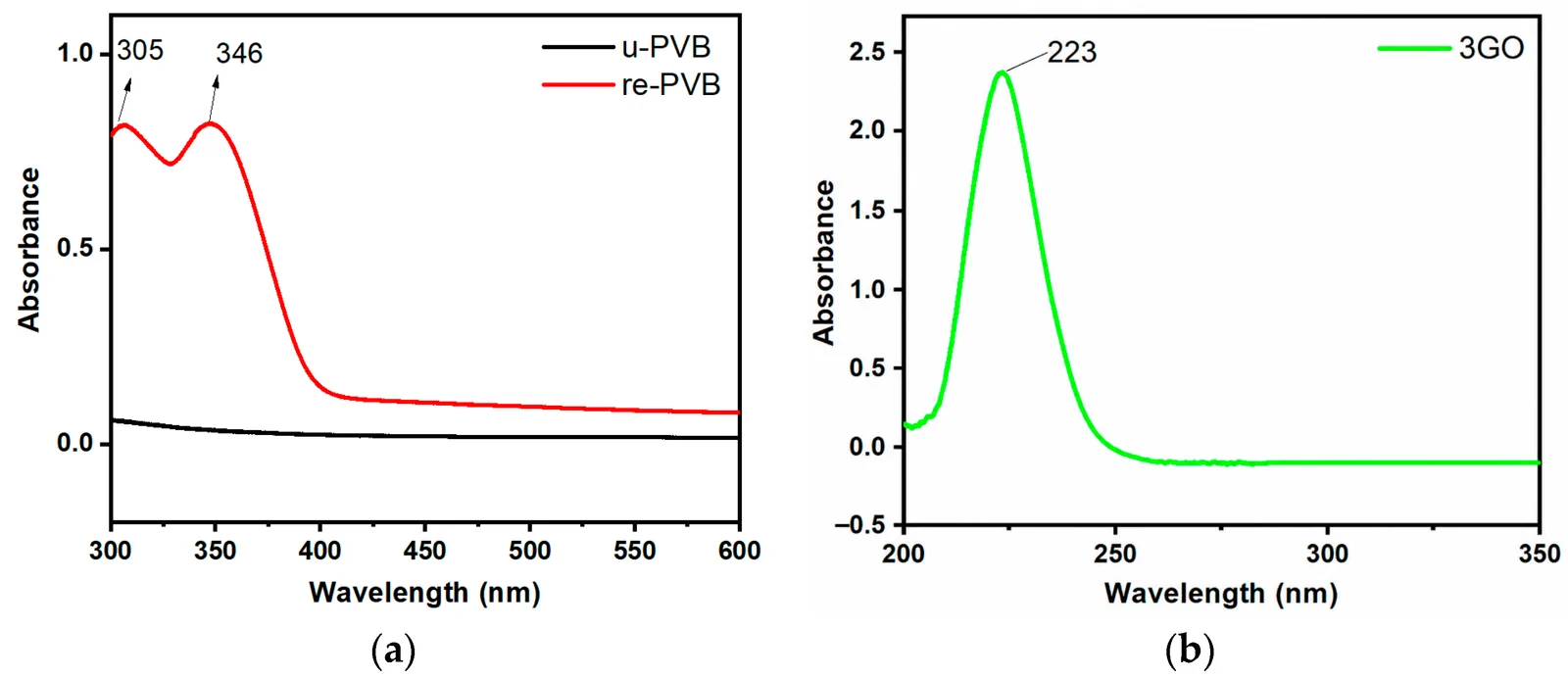
UV–Vis absorption spectra of (**a**) of u-PVB and re-PVB before artificial UV weathering and (**b**) pure 3GO plasticizer.

The superior weathering stability of re-PVB implicitly suggests the presence of residual additives capable of inhibiting photooxidative degradation. To investigate the origin of this enhanced UV stability, UV–Vis spectroscopy was performed on ethanol solutions of u-PVB, re-PVB and 3GO plasticizer. As shown in Figure 7a, u-PVB exhibited negligible absorption in the investigated UV-A region, consistent with the absence of stabilizing additives. On the other hand, re-PVB showed increased UV absorption at two distinct peaks (approximately 305 and 346 nm). The latter are characteristic absorption bands of a UV absorber, possibly of the benzotriazole type [38,44,45]. To exclude whether the included plasticizer in re-PVB could also contribute to the observed UV absorption, pure 3GO was also analyzed by UV–Vis spectroscopy. 3GO exhibited a strong absorption maximum at approximately 223 nm, while no absorption was observed in the tested UV-A radiation (340 nm); thus, no contribution in the UV stabilization of re-PVB is expected. 

The residual UV-absorbers identified in re-PVB, could be responsible for its superior UV stability. However, UV absorbers may not represent the only residual components of the original stabilization system that could still be present in re-PVB. PVB interlayer formulations also usually contain other stabilizing species, including hindered amine light stabilizers (HALSs) and antioxidants. According to the patent literature, the incorporation of HALSs and/or NOR-HALSs into stabilized PVB films can vary from concentrations of 0.005 to 1% *w*/*w* [12,13,15]. Therefore, residual HALSs are also expected since they can contribute crucially to the strong UV stability of a polymer by scavenging free radicals [34]. Moreover, the integration of the PVB interlayer between glass sheets may limit the loss of HALSs through leaching during its service life, potentially contributing to its retention in the retrieved material. In addition to the UV-absorbing species suggested by the UV–Vis results, the potential presence of residual HALSs in re-PVB was further investigated by ^1^H-NMR spectroscopy (Figure S3). Tinuvin 770 was analyzed as the reference HALS, since it is one of the most common ones reported in patents, and its spectrum was compared with those of re-PVB and u-PVB. Tinuvin 770 exhibited a characteristic multiplet centered at approximately 5.18 ppm, arising from the coupling of the methine proton in the –CH–O–C(O)– moiety with the axial and equatorial protons of the piperidine ring. In re-PVB, a multiplet peak is again observed, with some shifting (around 5.2 ppm) but it does not directly resemble the one from Tinuvin 770. On the contrary, in the u-PVB spectrum at the same ppm range, a doublet peak of much lower intensity is identified, which does not match the one from re-PVB nor from Tinuvin 770. The increased intensities and peaks of re-PVB compared with u-PVB at similar chemical shifts (at ca. 5.2 ppm) could represent the presence of HALSs of similar chemical structures but could not be unambiguously assigned to Tinuvin 770. In addition, the peaks identified at ca. 5.2 ppm for re-PVB are not reported in the literature in similar ^1^H-NMR spectra of plasticized PVB grade materials; thus, this signal could indicate the presence of an additive [1]. Ultimately, the ^1^H-NMR analysis did not provide conclusive evidence for the identification of residual HALSs in re-PVB. The absolute verification of the presence or identification of specific residual HALSs within the complex re-PVB formulation would require dedicated targeted and sensitive analysis, such as HPLC–MS, which could be the scope of another paper [17]. Nevertheless, considering the heterogeneous origin of re-PVB, which comprises PVB flakes derived from different laminated glasses and different interlayer formulations, the presence of residual HALSs in at least a fraction of the material cannot be excluded.

### 3.3. Effect of Phenolic Antioxidant Restabilization on the UV Weathering Stability of re-PVB

**Figure 8:**
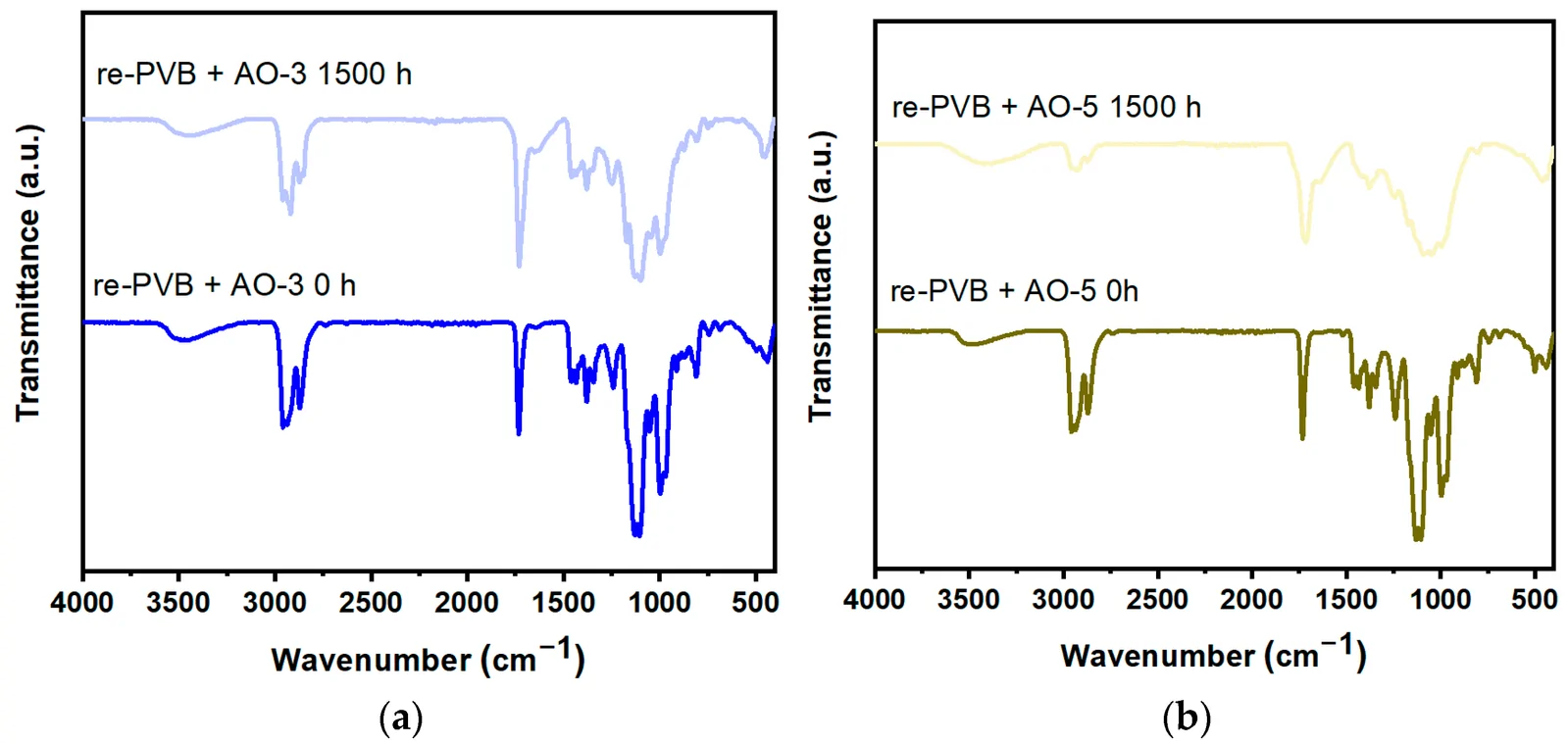
ATR-FTIR spectra of re-PVB restabilized with (**a**) AO-3 and (**b**) AO-5, before (0 h) and after (1500 h) artificial UV weathering.

The excellent UV stability of re-PVB was associated with the persistence of residual components from the original stabilization system. However, during the mechanical recycling of PVB, additional thermal stabilization was found to be mandatory to protect the polymer against thermo-mechanical degradation induced by melt reprocessing [33]. For that matter, the best performing phenolic antioxidants (AO-3 and AO-5), from our previous work, were incorporated in re-PVB at 0.3% *w*/*w*, so as to evaluate the joint performance of the AOs and the residual UV stabilization system against artificial accelerated weathering [33]. Unexpectedly, the presence of these phenolic antioxidants in re-PVB caused substantial deterioration in the material during UV weathering. As shown by the FTIR spectra (Figure 8a,b), substantial chemical changes in both the re-PVB + AO-3 and re-PVB + AO-5 formulations were observed after 1500 h of artificial UV weathering.

More specifically, the restabilized formulations exhibited changes typical to those observed during the photooxidative degradation of u-PVB, suggesting that the protective effect of the residual stabilization system was impaired upon addition of the antioxidants. The addition of AO-3 resulted in the increase in the carbonyl species (1740 cm^−1^) as well as the unsaturated structures (1600 cm^−1^) (Figure 8a), while in the case of AO-5, photooxidative degradation was even more chemically intense, resulting in the cleavage of the acetal ring at 900 cm^−1^ and 1100 cm^−1^ (Figure 8b). These spectral changes contrast strongly with the unchanged FTIR spectrum of untreated re-PVB after the same exposure period (Figure 5b) and show the extensive structural modification of the restabilized formulations during prolonged weathering. 

**Figure 9:**
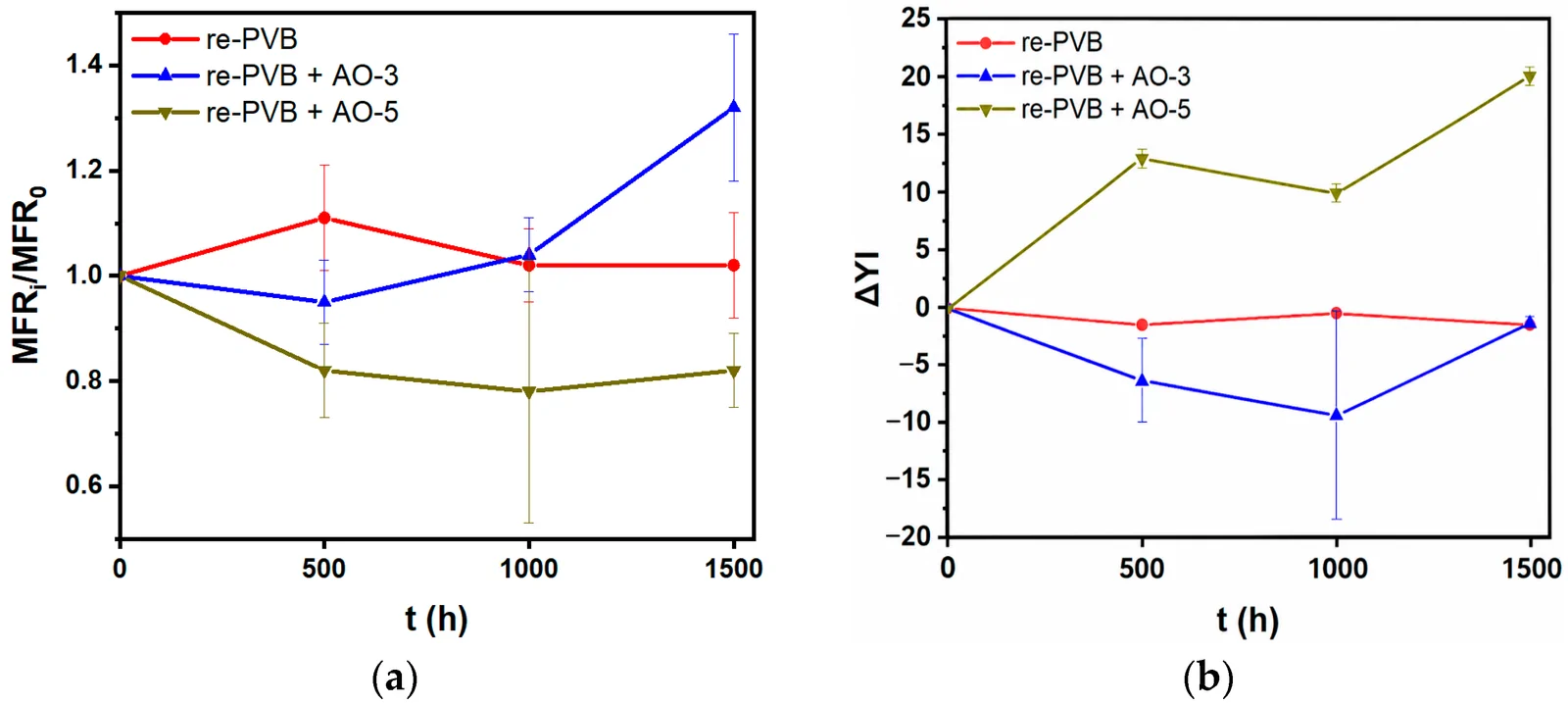
Changes in (**a**) normalized melt flow rate (MFR/MFR_0_) and (**b**) yellowness index (ΔYI) of untreated re-PVB and re-PVB restabilized with AO-3 and AO-5 during artificial UV weathering for up to 1500 h.

Further characterization showed that the restabilized samples also exhibited changes in melt flow behavior (Figure 9a). For re-PVB + AO-3, an increase in the normalized MFR of ca. 25% was observed after 1500 h, which may be associated with molecular weight reduction through chain scission. Conversely, re-PVB + AO-5 exhibited a slight decrease in the MFR during weathering, due to potential branching or crosslinking, since there were also solubility issues observed in that case (ranging from 4% to 13% *w*/*w*), which was not the case for the untreated re-PVB. Additionally, the macroscopic appearance of the MFR strands for the restabilized samples after weathering provided evidence for their degraded status (Figure S2c,d). In particular, re-PVB containing AO-3 or AO-5 exhibited intense browning in the strands, indicating the degradation of the melt processability of the material. The evolution of ΔYI revealed different color responses for the two formulations (Figure 9b). re-PVB + AO-3 did not show a significant increase in yellowness, but large variations (STD values) were observed at intermediate exposure times, emphasizing the different behavior. Regarding re-PVB + AO-5, there was a remarkable increase in ΔYI (ΔYI = 20), reaching substantial yellowing at approximately 1500 h. The degraded restabilized samples also exhibited incomplete dissolution in THF solvent, limiting the ability to reliably assess their intrinsic viscosity via dilute solution viscometry. The reduced solubility may be associated with degradation similar to u-PVB, potentially involving the formation of less-soluble degradation products or intermolecular structures such as branches or crosslinks. 

Although AO-3 and AO-5 were previously found effective in suppressing thermooxidative degradation during melt reprocessing [33], these results suggest that the addition of phenolic antioxidants (AO-3 and AO-5) facilitated an antagonistic effect against the residual stabilization system of re-PVB during artificial UV weathering. 

**Figure 10:**
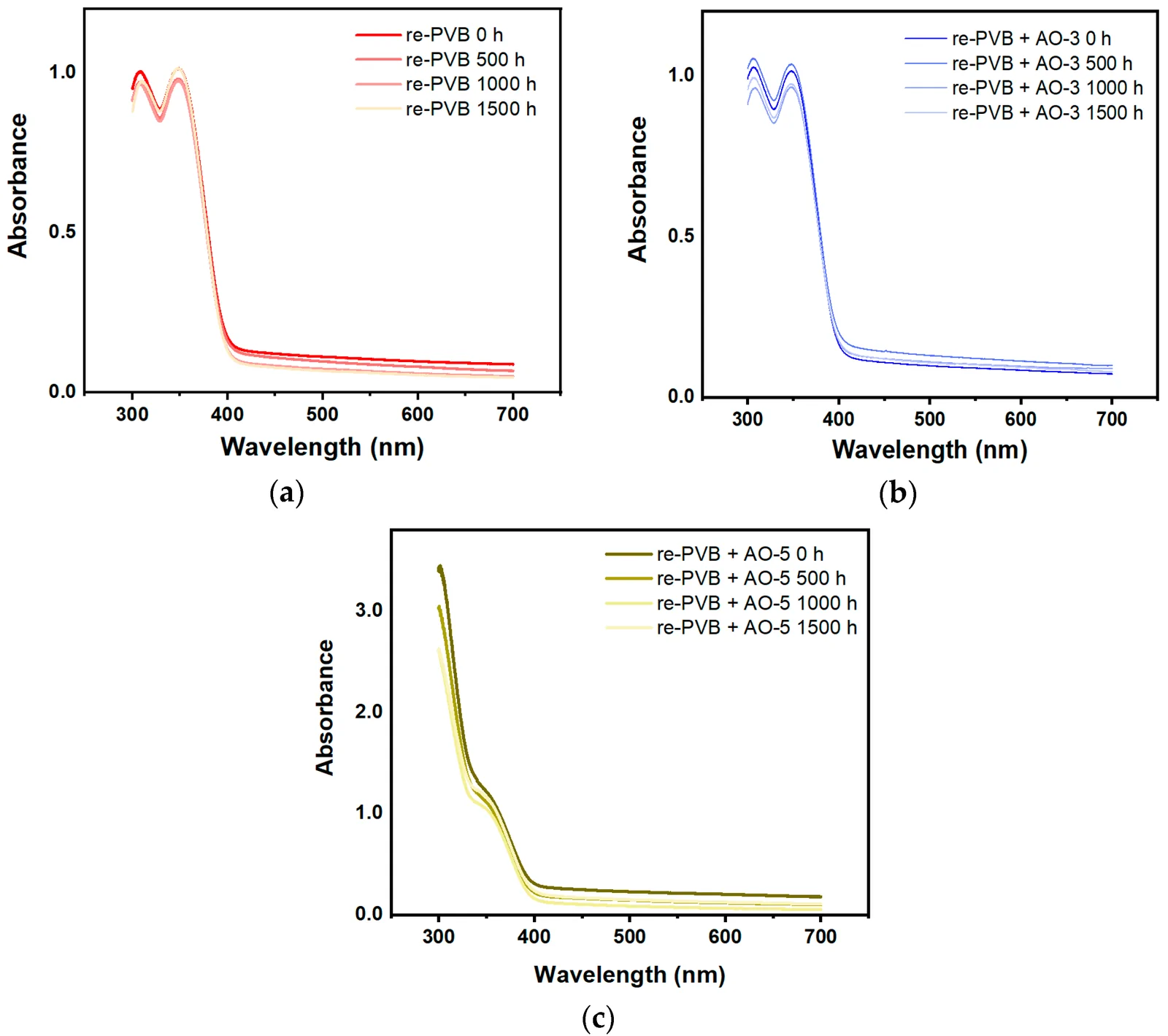
Evolution of UV–Vis absorption of re-PVB (**a**) and restabilized re-PVB with AO-3 (**b**) and AO-5 (**c**) formulations during artificial UV weathering up to 1500 h.

To determine whether the reduced photooxidative stability could be associated with a reaction of the residual UV-absorbing species, UV–Vis spectroscopy was performed on the formulations throughout the weathering period (Figure 10). The presence and the concentration of UV-absorbing species in the restabilized formulations remained largely unchanged after prolonged exposure, indicating that UV absorbers might not take part in any antagonistic reactions (Figure 10). The higher absorbance observed for the AO-5 formulation in the peaks at 305 and 346 nm is associated with the intrinsic molecular structure of AO-5 as a multifunctional phenol compound that contains UV-absorbing species of the 1,3,5-triazine group (Figure 1). However, this formulation’s absorbance also remained stable. In defense of this finding, UV absorbers generally act by absorbing incident UV radiation and dissipating the absorbed energy non-radiatively; thus, they are unlikely to interfere chemically in the stabilization path [28]. In this case, the antagonistic behavior may involve other components of the residual stabilization system and not UV absorbers, since they remain constant. 

One possible cause of the accelerated degradation in the restabilized formulations could be the formation of photoactive products derived from the degradation of phenolic antioxidants. The latter may undergo oxidation during processing or UV weathering, leading to conjugated or quinonoid species, some of which can contribute to yellowing and potentially participate in subsequent increased UV absorption [46,47,48]. However, the UV–Vis results did not provide evidence that new distinct absorption features attributable to antioxidant-derived chromophores developed during weathering. Consequently, this scenario is not considered probable. Another more plausible explanation of this behavior involves antagonistic interactions between the newly added phenolic antioxidants and components of the residual stabilization system inherited from the original PVB formulation [49]. More specifically, it has been reported that the introduction of phenolic antioxidants into a pre-existing stabilizer system may lead to antagonistic reactions with components such as HALSs, resulting in reduced performance [47,49,50]. The previous study by Beismann et al. demonstrated that, indeed, phenolic antioxidants can interfere with the HALS stabilization cycle by competing for radical species and disrupting nitroxyl radical regeneration, thus resulting in the useless consumption of both antioxidants and HALSs [47]. Although their study was conducted on polyolefins, the underlying stabilization mechanisms are expected to be relevant to PVB, causing the depletion of residual additives such as antioxidants and HALSs in re-PVB, thus reducing its overall UV stability. 

The fact that the incorporated phenolic antioxidants (AO-3 and AO-5) were previously effective under thermooxidative reprocessing conditions but reduced the photooxidative stability of re-PVB during UV weathering suggests that the observed antagonistic behavior is strongly dependent on the degradation conditions (UV radiation) and the generated radicals. Under UV irradiation, additional radicals are generated through photochemical processes, including hydroperoxide photolysis and reactions of light-absorbing species, which may consequently increase the interactions between the different stabilizing components. On the contrary, thermooxidative degradation occurring during melt reprocessing, such as during repeated extrusion cycles or prolonged residence in a Banbury-type internal mixer, involves thermally initiated radical reactions. The thermal degradation of PVB has been proposed to involve H-abstraction from tertiary carbon sites in the polymer, resulting in the formation of carbon-centered polymer radicals (R*) [51]. Although radical species are therefore also generated under thermooxidative conditions, the relatively short duration and the high temperatures associated with melt reprocessing may limit the extent of intermediates such as hydroperoxides. Under the reprocessing conditions investigated in our previous work, degradation was comparatively limited, with no pronounced modification of the PVB chemical structure in u-PVB, and the main FTIR change was a moderate increase in the intensity of the carbonyl region in the range of 1600–1800 cm^−1^ [33]. In such an environment, phenolic antioxidants can effectively perform by interrupting the oxidation cycle through H-atom donation to the radical species, thereby limiting propagation reactions [28]. Consequently, under thermooxidative conditions, the protective action of phenolic antioxidants may dominate over any potentially unfavorable interactions. In contrast, under photooxidative conditions, antagonistic interactions between the phenolic antioxidants and the pre-existing stabilization system could arise, resulting in reduced UV stability.

## 4. Conclusions

High-quality retrieved PVB (re-PVB) from laminated glass waste can retain substantial functionality after its first service life and presents exceptional resistance to subsequent photooxidation. On the contrary, neat unplasticized PVB severely degraded during artificial UV weathering, exhibiting chemical changes, molecular weight reduction, increased melt flow, and yellowing. The UV–Vis analysis revealed the persistence of UV-absorbing species in re-PVB, indicating that the components of the original stabilization system remain present and functional after service and retrieval of the material. The preservation of these additives may be favored by the reduced service life of a broken/substituted laminated glass or the near absence of oxygen within the laminated structure that delays the photooxidation of PVB. The restabilization of re-PVB with phenolic antioxidants was previously found to be effective and mandatory during melt reprocessing, although these additives were not expected to provide additional protection against UV weathering. Instead, the restabilized formulations with AOs exhibited severe deterioration, revealing an apparent antagonistic effect between the newly introduced antioxidants and the residual stabilization system, possibly HALSs. From a waste-valorization perspective, these findings emphasize that effective sorting (selectivity in the laminated glass waste) can result in PVB with a residual UV additive load that is still active. Thus, re-PVB is considered a valuable waste without the need of UV re-additivation, therefore contributing to the circular plastic economy. In other words, high-quality re-PVB appears to be a valuable waste stream with potential for reuse without additional UV stabilization, provided that appropriate sorting and recovery procedures are employed. However, the need for thermal restabilization during melt reprocessing should be considered separately.

## Figures and Tables

**Table 1:** Thermogravimetric analysis (TGA) results for u-PVB, re-PVB, and 3GO. T_5_% denotes the temperature at 5% mass loss, T_d_ is the temperature at the maximum rate of mass loss determined from the DTG curve, and residue is the remaining mass at 600 °C. Values are reported as mean ± standard deviation.

Samples	*T*_5%_ (°C)	1st Mass Loss (% *w*/*w*)	*T*_d1_ (°C)	*T*_d2_ (°C)	Residue (% *w*/*w*)
		Plasticizer Evaporation		PVB Decomposition	
u-PVB	329.1 ± 13.1	-	-	391.1 ± 0.4	1.0 ± 1.0
re-PVB	261.7 ± 4.5	39.4 ± 4.4	313.8 ± 1.9	388.7 ± 4.5	2.6 ± 0.1
3GO	187.5 ± 6.2	98.4 ± 1.2	272.9 ± 24.0	-	1.6 ± 1.2

**Table 2:** Initial yellowness index (YI) of u-PVB, re-PVB, and commercial PVB (Eastman Saflex RB41).

Samples	YI
u-PVB	28.49 ± 0.86
re-PVB	40.41 ± 0.22
Benchmark PVB grade	33.91 ± 0.04

**Table 3:** Weight-average molecular weight (Mw, PS-equivalent), number-average molecular weight (Mn, PS-equivalent) and polydispersity index (PDI) obtained by GPC, intrinsic viscosity ([*η*]) determined by DSV, and melt flow rate (MFR) of u-PVB and re-PVB.

Samples	Mw (g/mol)	Mn (g/mol)	PDI	[*η*] (dL/g)	MFR (g/10 min)
u-PVB	463,000	209,000	2.21	1.58 ± 0.01	0.55 ± 0.02 *
re-PVB	507,000	170,000	2.96	2.00 ± 0.10	2.47 ± 0.15 **

* Measured with 10 kg at 190 °C, ** measured with 2.16 kg at 190 °C.

## Data Availability

The original contributions presented in this study are included in the article/Supplementary Materials. Further inquiries can be directed to the corresponding authors.

## Supplementary Materials

The following supporting information can be downloaded at https://www.mdpi.com/article/10.3390/ma19214751/s1, Figure S1: Macroscopic appearance of (a) u-PVB, (b) re-PVB, (c) re-PVB + AO-3, and (d) re-PVB + AO-5 specimens during artificial UV weathering for up to 1500 h. Specimens are shown from left to right at 0, 500, 1000, and 1500 h of exposure; Figure S2: Macroscopic appearance of MFR strands obtained from (a) u-PVB, (b) re-PVB, (c) re-PVB + AO-3, and (d) re-PVB + AO-5 after artificial UV weathering for 0, 500, 1000, and 1500 h; Figure S3: Expanded ^1^H-NMR spectra of Tinuvin 770, u-PVB, and re-PVB (CDCl3 solvent) in the 4.7–5.7 ppm region, showing the characteristic Tinuvin 770 resonance around 5.18 ppm and the corresponding spectral region of the PVB samples.
