# Supplementary material for: Artificial UV Weathering of Retrieved Poly(vinyl butyral) Interlayer from Laminated Glass Waste: Effects of Residual Additives and Restabilization with Phenolic Antioxidants
Source: Materials (Basel). 2026 Nov 9;19(21):4751. doi: 10.3390/ma19214751 (PMC13765428; doi:10.3390/ma19214751)
Supplement: Supplementary file 1 [file materials-19-04751-s001.zip › materials-4665136-supplementary.pdf]

materials
MDPI

Supplementary Information

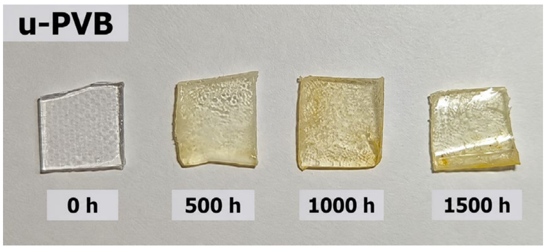

a

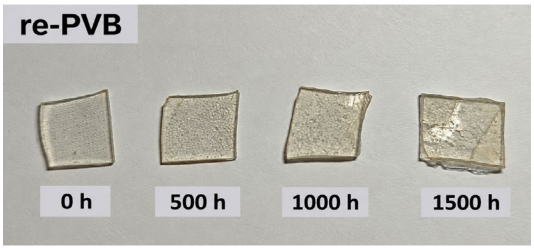

b

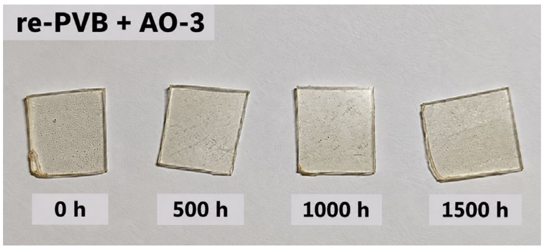

c

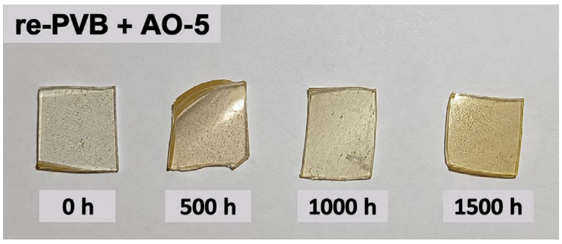

d

Figure S1: Macroscopic appearance of (a) u-PVB, (b) re-PVB, (c) re-PVB + AO-3, and (d) re-PVB + AO-5 specimens during artificial UV weathering for up to 1500 h. Specimens are shown from left to right at 0, 500, 1000, and 1500 h of exposure.

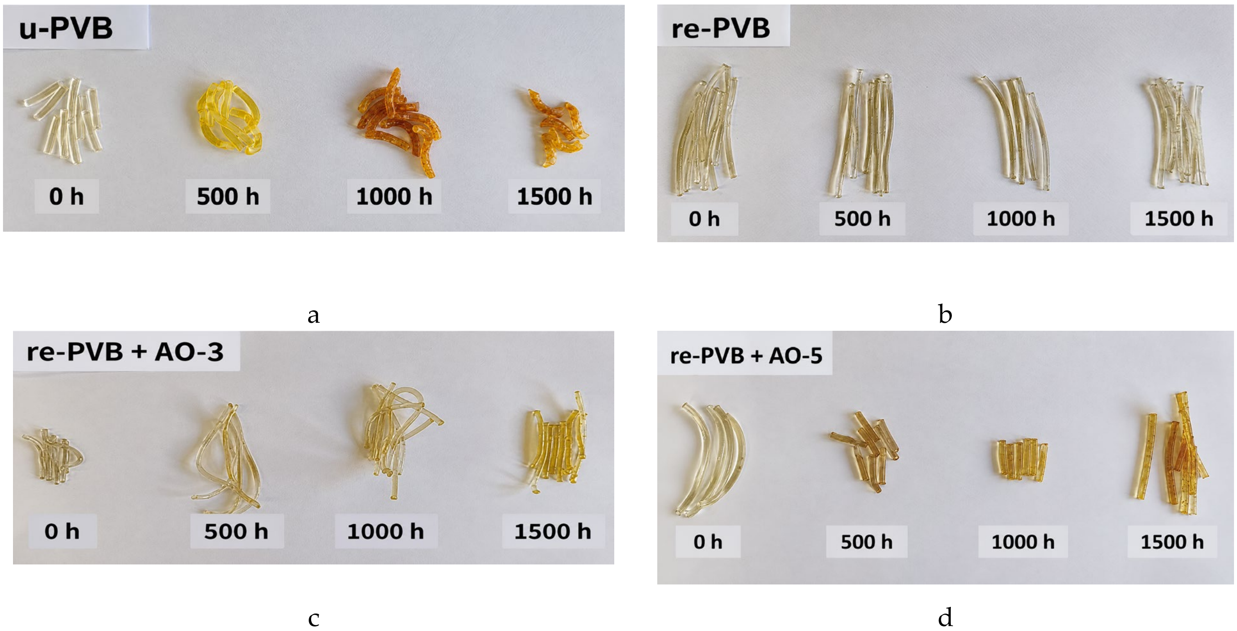

Figure S2: Macroscopic appearance of MFR strands obtained from (a) u-PVB, (b) re-PVB, (c) re-PVB + AO-3, and (d) re-PVB + AO-5 after artificial UV weathering for 0, 500, 1000, and 1500 h.

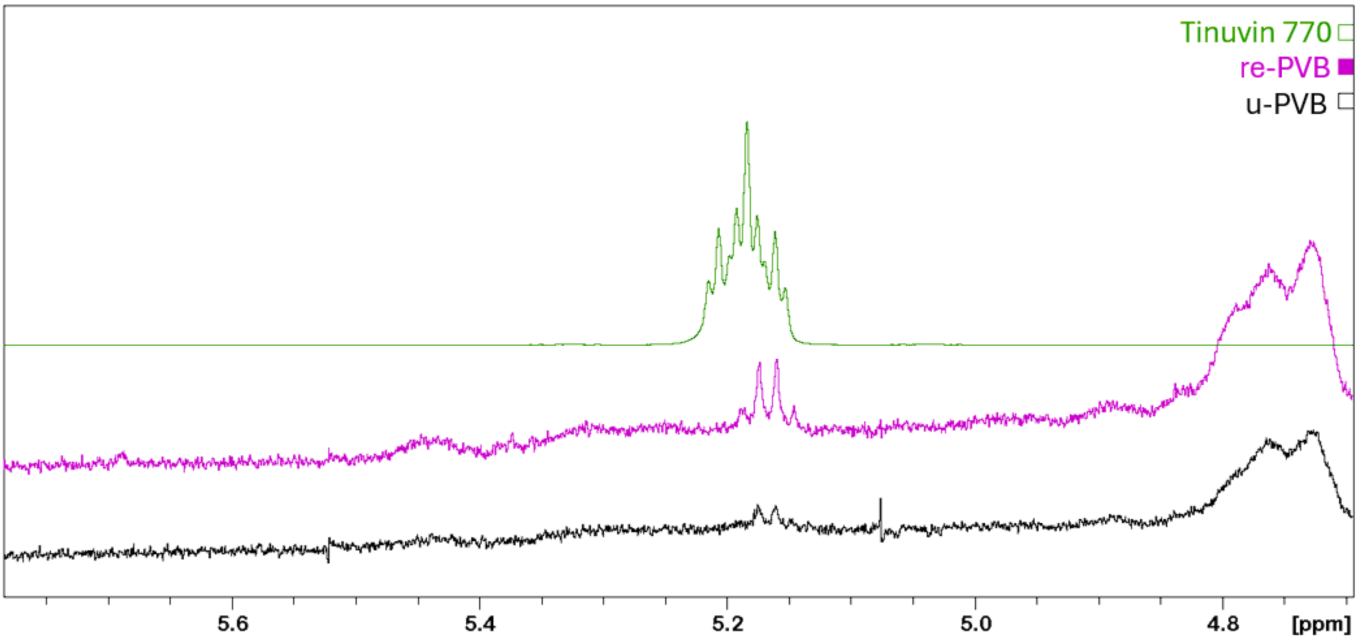

Figure S3: Expanded ^{1}H-NMR spectra of Tinuvin 770, u-PVB, and re-PVB (CDCl3 solvent) in the 4.7–5.7 ppm region, showing the characteristic Tinuvin 770 resonance around 5.18 ppm and the corresponding spectral region of the PVB samples.
